# Why Employees Experience Burnout: An Explanation of Illegitimate Tasks

**DOI:** 10.3390/ijerph19158923

**Published:** 2022-07-22

**Authors:** Chenhui Ouyang, Yongyue Zhu, Zhiqiang Ma, Xinyi Qian

**Affiliations:** School of Management, Jiangsu University, Zhenjiang 212013, China; 2111910013@stmail.ujs.edu.cn (C.O.); mzq@ujs.edu.cn (Z.M.); 15358065701@163.com (X.Q.)

**Keywords:** illegitimate tasks, job burnout, psychological entitlement, collective climate

## Abstract

Among the many workplace stressors, a new type of stressor has been identified: illegitimate tasks. This newly identified type of stressor refers to work tasks that do not meet employee role expectations and constitute a violation of professional identity. To investigate illegitimate tasks’ mediating mechanisms and boundary conditions on job burnout, we examined a cross-level first-stage moderated mediation model with the collective climate as a moderator and psychological entitlement as a mediator. Grounded in the job demands–resources model (JD-R) and justice theory, the current study uniquely posits that illegitimate tasks can lead to burnout by way of psychological entitlement; however, this effect is less where collective climate is higher. Data were collected from 459 employees on 89 teams at enterprises in China. The results of the analysis, using HLM, MPLUS and SPSS revealed that illegitimate tasks stimulated employees’ psychological entitlement and led to job burnout. While employees’ psychological entitlement played a partially mediating role between illegitimate tasks and job burnout, a collective climate could weaken the stimulating effect of illegitimate tasks on employees’ psychological entitlement and then negatively affect the mediating effect of psychological entitlement between illegitimate tasks and burnout. The study reveals the antecedents of burnout from the perspective of job tasks and psychological entitlement, offers practical insight into the mechanism of illegitimate tasks on employee job burnout and recommends that organizations develop a collective climate to reduce employees’ psychological entitlement and job burnout for steady development of the enterprise.

## 1. Introduction

The problem of job burnout is becoming increasingly prevalent worldwide [1]. After COVID-19 broke out in 2019, people’s perceptions and attitudes toward work underwent some changes, and the issue of job burnout became urgent as individuals reexamined the meaning and value of work in light of health concerns [2]. Burnout is generally conceptualized as a chronic stress syndrome, including chronic feelings of exhaustion, negative attitudes toward work (cynicism), and reduced professional efficacy [3,4]. Job burnout can damage employees’ physical and mental health [5], reduce their performance [6], and lead to negative workplace behaviors and safety accidents [3,7]. To alleviate or prevent a series of negative effects of burnout, it is necessary to focus on its causes. Therefore, some researchers point out that the key focus of burnout research is to explore its influencing factors [8].

Currently, the literature on burnout mainly follows the job demands–resources model (JD-R), which focuses on personality factors related to internal resources, organizational factors related to external environmental resources, and work factors related to job requirements. Personality factors include self-esteem [9], personality traits [10], psychological capital [11], etc.; organizational factors cover organizational culture [12], organizational climate [13], leadership style [14], etc.; work factors involve job stress [15], role conflict and role overload [16], workplace bullying [17], etc. In general, related studies have been continuously devoted to finding and verifying the factors that lead to employee job burnout, and rich research results have been obtained. The omnipresent view is that burnout is intrinsically associated with work factors and secondarily associated with personality factors and organizational factors [18]. With the volatility and uncertainty of the external environment, the need for continuous adaptation within the organization has led to the continuous emergence of new job characteristics in the work environment, making it pressing and significant to continuously explore the antecedents of burnout from the perspective of work factors.

Research in recent decades has revealed that burnout is often the result of high job demands—aspects of the job that require sustained physical, emotional, or cognitive effort [19]. Illegitimate tasks are just such a high job demand. Illegitimate tasks refer to work tasks that do not meet employee role expectations and constitute a violation of professional identity [20]. The issuance of illegitimate tasks poses a threat to rational resource allocation that cannot be underestimated. As a special kind of job stressor, its execution and completion come at the cost of the internal depletion of employees’ positive psychological resources, which can have a series of negative effects on employees, including burnout [21,22,23]. Although the literature shows that illegitimate tasks increase the risk of burnout, research still lacks a comprehensive understanding of the mediating effects between illegitimate tasks and burnout.

Justice theory is often used to explain the impact of illegitimate tasks. Employees may perceive that decisions about illegitimate task assignments are made in an inequitable manner and experience disrespect, perceived threats to their professional identity, and a sense of effort–reward imbalance [24]. Based on the injustice characteristic of illegitimate tasks, existing studies have explored the association between illegitimate tasks and employees’ negative emotions and job satisfaction [25,26], while neglecting psychological entitlement, a widespread phenomenon in the workplace. When individuals believe they are not receiving the results they deserve, such as equal treatment and respect, they develop a perception of being entitled to preferential treatment; that is, psychological entitlement [27]. Employees with psychological entitlement tend to have inflated self-perceptions; they focus on getting and neglect giving and are more likely to see illegitimate tasks as an excessive job requirement. Meanwhile, they are very sensitive to psychological contract violation, more likely to perceive the loss of resources, and demand many psychological resources for self-regulation [28]. According to JD-R theory, high job demands and loss of resources trigger burnout. Therefore, employees with high psychological entitlement may be more prone to job burnout. However, few of the current studies related to illegitimate tasks and burnout have focused on psychological entitlement as a psychological phenomenon detrimental to employees’ psychological well-being and performance. The present study aims to fill this research gap by attempting to introduce psychological entitlement as a psychological mechanism by which illegitimate tasks act on employee burnout.

**Figure 1:**
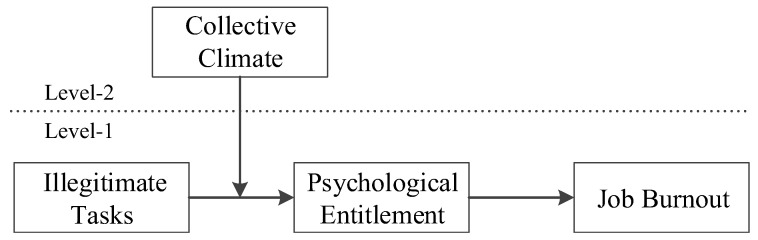
The theoretical conceptual model.

Exploring illegitimate tasks and psychological entitlement from the perspective of justice theory needs to consider the influence of employees’ work values and traditional cultural concepts [29]. Justice perception, as a subjective feeling, can vary in organizations with different cultural climates. Currently, in the context of globalization, Chinese organizations have both traditional and modern concepts under the collision of Western and traditional cultures. Traditionalism is rooted in Confucianism and emphasizes collectivism, where team needs take precedence over individual needs, while modernity focuses on self-centered individualism [29]. The integration of traditionality, characterized by high collectivism, into elements of modernity tends to result in organizations with varying levels of collective climate, and employees exhibit cognitive schemas, affective responses, and behavioral expressions that are compatible with it [30]. At higher levels of collective climate, employees have higher attachment to the work group, level of commitment to the organization, and willingness to contribute [31]. Thus, they may change their sense of identity violation, be more accepting of illegitimate tasks and let go of strong perceptions of injustice and psychological entitlement. In other words, the cultural climate shapes individuals’ way of thinking and the process by which they are influenced by illegitimate tasks [32]; thus, individuals develop psychological entitlement and burnout that may be enhanced or weakened by the collective climate. This study further introduces collective climate as a moderating variable and constructs a cross-level moderated mediation theoretical model (as shown in Figure 1) based on justice theory and the JD-R model. This process enables the in-depth exploration of the mediating paths and boundary conditions of employee burnout triggered by illegitimate tasks and provides more comprehensive insights for managing employee burnout and promoting employee occupational health.

## 2. Literature Review and Hypothesis Development

### 2.1. Illegitimate Tasks and Employee Job Burnout

The concept of illegitimate tasks is based on the stress-as-offense-to-self theory, which states that individuals perceive threats to their self-identity and image as a source of stress [33]. In organizations, individuals usually play different occupational roles and follow a clear set of behavioral norms or guidelines based on occupational characteristics and job design, i.e., role expectations [34]. When a job task exceeds or contradicts the role expectations held by the employee, the task is implicitly offensive to the employee’s professional role and is therefore considered to be an unregulated job task. There are two facets of illegitimate tasks, which we call unreasonable tasks and unnecessary tasks [20]. In layman’s terms, unreasonable tasks are tasks that the individual believes should not be his or her responsibility, which are either beyond the scope of his or her duties, conflict with professional status, do not reflect professional identity, or put the individual in an awkward situation. Unnecessary tasks are tasks that are meaningless and can be avoided and sometimes may exist only because of the leader’s personal preference [20]. In short, unreasonable tasks are tasks that are not appropriate to ask from a specific person, unnecessary tasks are tasks that should not have to be carried out at all, and together they constitute the two dimensions of illegitimate tasks. Illegitimate tasks essentially stem from the inappropriate use of authority by leaders, and leaders issuing illegitimate tasks can send signals of disrespect and devaluation to employees [35], which is highly likely to lead to negative work attitudes and behaviors. Studies have found that illegitimate tasks can lower employees’ self-esteem [36], make employees feel unappreciated by the organization or leader [25], reduce job satisfaction and damage the formation of individual occupational well-being [21,37]. At the same time, as a job stressor, it can have a negative impact on job commitment, job identity, and job meaning [22,38]. Some recent studies have also indicated that illegitimate tasks pose a threat to employees’ professional identities and can trigger burnout [21,23,26]. The findings of related studies provide a good reference for this study.

According to the JD-R model, psychological stress occurs when resources (e.g., social support, positive perceptions and emotions) become insufficient to meet (high) demands (e.g., subjective fatigue, reduced focus of attention, and redefinition of task requirements), which ultimately may lead to resource depletion and the development of burnout when resources are not timely replenished [2,39]. This study argues that the impact of illegitimate tasks on employee job burnout can be explained by two attrition paths: increasing work demands and decreasing work resources. On the one hand, illegitimate tasks have the typical characteristics of high effort–low reward [34]. Illegitimate tasks exceed employees’ role expectations, are not related to employees’ core work and are additional work demands that require more time and effort than normal work [40]. This will cause employees to feel exhausted, want to disengage, and lose enthusiasm for their work, that is, experience job burnout. On the other hand, illegitimate tasks imply social messages of devaluation and disrespect from superiors, which pose a threat to employees’ self-esteem and professional identity and can trigger strong negative emotional reactions [41]. In the face of illegitimate tasks, employees need to use many cognitive resources for emotional regulation and psychological adjustment and expend energy resources and individual characteristic resources to perform the tasks, which leads to the damage of reserve resources. The employee eventually becomes “resource poor” and then shows the work state of job burnout [25]. In short, illegitimate tasks increase work demands while decreasing work resources, bringing about an imbalance between demands and resources derived from work. When demands exceed resources, fatigue occurs; if this imbalance is maintained over time, fatigue becomes chronic and, finally, burnout occurs [2]. In line with the literature discussed above, we pose the following hypothesis:

**Hypothesis** **1.** *Illegitimate tasks have a positive effect on job burnout*.

### 2.2. The Mediating Role of Psychological Entitlement

Psychological entitlement is a subjective belief or perception that individuals feel entitled to certain preferential treatment and exemption from certain social responsibilities [42]. Harvey and Martinko further defined psychological entitlement in the workplace by arguing that at the core of psychological entitlement in the workplace is an employee’s desire for special treatment and rewards that are not related to actual performance [43]. Psychological entitlement can be considered a stable personality trait or a psychological state that can be activated by specific individuals or situational factors [27,42,43]. Regarding the factors that stimulate employees’ psychological entitlement, specific leadership style, commonality of creativity, etc., will increase employees’ psychological entitlement by providing special working conditions and making them feel preferentially treated [44,45]. In addition to this motivational path, some studies have also indicated that psychological entitlement comes largely from the absence or deviation of a sense of inner justice [46]. In other words, inequitable treatment can stimulate employees’ psychological entitlement [27]. These findings provide some basis for this study to speculate on the possible link between illegitimate tasks and psychological entitlement.

If the illegitimate task is essentially a demotion, such as asking a nurse to clean a toilet (task beyond the scope of duties) or a senior lawyer to take minutes in a meeting (task in conflict with professional status), then employees may perceive their skills or abilities as undervalued and disrespected. If the illegitimate task is presented in an upward manner, such as requiring a group leader to complete a core task of a department head (task in conflict), then the employee may not be able to complete the task due to lack of relevant competencies and experience, which is undoubtedly an overt failure that can seriously dampen the employee’s perceived self-worth [47]. Both disrespect and impaired self-evaluation can make employees believe that they have taken an undeserved loss and deserve more entitlements [48].

In addition, completing illegitimate tasks either requires compressed work time on core tasks and the risk of dereliction of duty or additional time and effort and increased workload. In both cases, employees develop the psychological perception of an effort-reward imbalance and thus feel that they are entitled to some form of compensation, which is referred to as psychological entitlement [44]. Therefore, this study argues that illegitimate tasks can be considered an inequitable event that increases employees’ psychological entitlement by offending their positive self-concept and demanding more labor than the job requires. Thus, the following hypothesis is proposed:

**Hypothesis** **2.** *Illegitimate tasks have a positive effect on employees’ psychological entitlement*.

This study argues that employees with high psychological entitlement may exhibit higher levels of job burnout. From the perspective of job demands, employees with high psychological entitlement possess an inflated self-perception that their efforts are more valuable than what they receive in return. However, this expectation is not matched with actual competence and performance and is often difficult to achieve [49]. This leads employees to feel that work-based social exchange is not reciprocal and creates the perception that work demands exceed expectations [50]. The perception of inequity brought about by this gap between expectations and reality can significantly reduce employees’ motivation to work hard, making them more frequently prone to job withdrawal and higher levels of job burnout. Employees with high psychological entitlement have a strong self-serving bias. They are self-centered and are prone to attribute unfavorable results to outside influences. They may be disappointed and dissatisfied with their leaders and even the organization. They may experience strong negative emotions and frustration [49,51], while they tend to have a weaker self-concept and need constant self-affirmation [52], thus requiring more psychological resources for self-regulation. This continuous depletion of resources further induces employee burnout.

The assignment of illegitimate tasks does not meet employees’ role expectations and may even cause identity violation, which leads to a stronger sense of relative deprivation and injustice when employees compare their role expectations with their job status and work effort with gain. Employees may believe that such tasks should not be assigned to them or would be better handled in other ways, generating the perception of “harming their own rights and interests” [33]. Therefore, regardless of performance, they expect to be entitled to some compensation or exempt from some responsibilities, forming a higher psychological entitlement. Employees with high psychological entitlement will have unrealistic expectations and will not be satisfied even with rewards commensurate with their efforts and abilities. They will feel unappreciated and constantly fall into a vortex of self-doubt and self-inflation [49]. As they experience higher work demands and consume more psychological resources for self-regulation, these employees will experience emotional exhaustion, cynicism, and low achievement symptoms. That is, illegitimate tasks will contribute to job burnout by stimulating these employees’ psychological entitlements. In summary, this study proposes the following hypothesis:

**Hypothesis** **3.** *Psychological entitlement plays a mediating role between illegitimate tasks and employee job burnout*.

### 2.3. The Moderating Role of Collective Climate

Collective climate refers to a value shared by organizational members that focuses on collective interests and goals and follows collective norms [53]. A strong collective climate is one of the characteristics of Chinese culture. Being influenced by traditional culture, individuals generally hold a dependent self-concept and place more importance on interpersonal care, human adaptation to the environment, and harmonious interpersonal relationships, resulting in a higher level of collectivist orientation [54]. When there are more employees with a collectivist orientation on a work team, it has a high collective climate [55]. Against the backdrop of “rising calls” for more localized research, an increasing number of scholars have begun to focus on the possible role of cultural factors, such as collectivism, in shaping individual attitudes and behaviors [56]. A high collectivist culture in a country makes people form attachments to in-groups through socialization processes. When individuals enter an organization, they translate these attachments into attachments to the work team to which they belong, which leads to higher levels of organizational commitment, more attention to organizational goals and interests, and a willingness to contribute to the organization [31]. This organizational interest-first mindset, which reflects the extent to which individuals are willing to adapt to the organizational environment and maintain interpersonal connections, may play a buffering role in the process of illegitimate tasks triggering negative attitudes and behaviors in employees.

Specifically, the boundaries between the individual and the organization are relatively blurred in a highly collective climate. Individuals recognize their group identity more and view themselves as a part of the work team to which they belong. Employees are more loyal and emotionally attached, prefer to consider problems from a holistic perspective and are willing to devote themselves to the team’s interests [31]. Consequently, they may hold different interpretative schemata in the face of the issuance of illegitimate tasks and have higher tolerance and acceptance of the conflicting role expectations and threats to their professional identities posed by illegitimate tasks. Even when faced with the stimulus of illegitimate tasks, employees tend to selectively interpret them in the context of the bigger picture based on positive information cues in the environment [57], weakening the perceived unfairness brought about by illegitimate tasks, reducing the level of psychological entitlement and weakening self-interested thoughts and behaviors. In addition, the “harmonious interpersonal perspective” promoted by the collective climate leads to close and harmonious relationships among team members who are willing to support their leaders and help their colleagues [58]. Even if they do what others should do, they will consider it from the perspective of “helping others” and “social relations” and pay less attention to personal gains and losses, thus diminishing psychological entitlements. Based on the above analysis, the following hypothesis is proposed.

**Hypothesis** **4.** *A collective climate negatively moderates the positive relationship between illegitimate tasks and employees’ psychological entitlement. The higher the collective climate is, the weaker the positive relationship between illegitimate tasks and employee psychological entitlement*.

Furthermore, organizations and teams in Chinese cultural contexts are often viewed as families in a broad sense, while the climate in a team is a consensus among team members about the team norms to be followed [59]. Therefore, employees’ attitudes and behaviors are strongly influenced by the team climate [58]. Employees undertaking illegitimate tasks, whether unreasonable or unnecessary, formally contribute to the team at the expense of personal resources and individual interests [34]. In a high collective climate, employees contributing to the team usually gain respect, trust, and recognition from team members [57], facilitating the establishment of high-quality team member relationships and satisfying individuals’ intrinsic need for relationships. Thus, a collective climate alleviates employees’ psychological entitlements and help them mobilize more surplus energy to overcome the depletion of resources, thus alleviating the symptoms of emotional exhaustion, cynicism, and low achievement caused by illegitimate tasks. As a result of a high collective climate, employees are no longer primarily focused on self-development and achieving personal goals but rather are more attentive to the needs and interests of the group. Despite receiving illegitimate tasks, their arousal of psychological entitlement is diminished, and consequently, the level of job burnout will be improved. Accordingly, the following hypothesis is advanced:

**Hypothesis** **5.** *Collective climate negatively moderates the mediating role of psychological entitlement between illegitimate tasks and employee job burnout, and the higher the collective climate is, the weaker the mediating role of psychological entitlement*.

## 3. Materials and Methods

### 3.1. Sample and Data Collection

We obtained consent to participate in the study from 25 companies located in the Yangtze River Delta region, all of which had established relationships with the research group at an early stage. These 25 companies belong to four representative industries, including construction (5), health care (4), manufacturing (9), and production and supply of electric power and heat (7) [7]. The survey was conducted from October to December 2020. This current study was aware of non-response bias that could have affected the response rate of the survey. This is because the survey was done during the peak of COVID-19 pandemic where there were several restrictions and lockdowns in China. Also, some of the survey items could be intimidating, especially, items on the illegitimate tasks and burnout. According to Berg et al., respondents who feel intimidated by research questions are likely to decline participation, thereby reducing a study’s response rate [60]. Again, Smironva et al. indicated that some respondents will definitely not participate in the survey even after receiving the survey questionnaires [61]. Therefore, to mitigate the impact that non-response bias could have on the study’s overall response rate, we used the variable to sample ratio technique to increase the response rate. The variable to sample ratio suggests that a proposed sample size selection should be based on the ratio of respondents to items [62,63]. The ratio is expressed as *N*: *p*. The *N* represents number respondents while the *p* represents number of items. Sample suggestions for the variable to item ratio include 3:1, 6:1, 10:1, and even 20:1. In this current study where we have a total of 40 items (35 items from measurement scales, 5 items for measuring demographic and work-related variables), a variable to sample ratio of 10:1 approach was used. The 10:1 ratio implies that a sample size of 400 could have been enough for this survey analysis. However, we distributed 500 questionnaires and obtained 459 valid responses. This approach has the tendency of mitigating any inaccuracies non-response biases could account in a survey analysis [64]. The selection method we used in selecting the sample work teams was stratified sampling. We asked these 25 companies about their company size, the number of work teams, and then calculated the average work team size for these 25 companies to be 5.4. In order to distribute 500 questionnaires, we needed to survey at least 92 work teams. We selected 92 work teams from these 25 companies using a simple random sampling technique. The number of work teams sampled for each company was determined by the number of work teams in that company. 

During the survey, questionnaires for each team member were prepared, placed in separate envelopes and mailed to the contact person, who was asked to distribute the questionnaires and collect the completed ones. To diminish participants’ concerns about possible retaliation from their leaders, we asked each participant to seal the envelope immediately after completing the questionnaire to ensure that no colleagues or leaders would see their completed questionnaires. We also asked the contacts to remind the participants to seal the envelope when collecting the questionnaires. To control the quality of the questionnaires, the number of questionnaires distributed to each work team was limited to 3~10. After filtering and deleting invalid questionnaires that were not completely filled out or selected the same options for all items or otherwise obviously not answered carefully, a total of 459 valid research results were finally obtained from 89 work teams, with a valid recovery rate of 91.80%. The response rate (i.e., >70%) further reduced the nonresponse bias [65]. Despite the non-response bias, it was felt that the sample provides the mixture of employees from various departments and the number of responses is reasonable to undertake meaningful analysis [13].

The average number of members of 89 work teams was 5.16 with a range of 4–6. Work teams belonging to state-owned enterprises contributed the most teams with 44 (49.44%), followed by private enterprises, with 22 (24.72%), and the remaining 23 subjects worked for international joint ventures and others. Among the 459 subjects, the ratio of males to females was similar, with 205 males (44.66%) and 254 females (55.34%). The age distribution was relatively wide, among which the 26- to 30-year-old cohort was the most common, accounting for 110 participants (23.97%), followed by over 40 years old, with 101 people (22.00%); more than half of the participants had a bachelor’s degree, with 264 people (57.52%); and 202 people had worked in the company for more than 5 years, accounting for 44.01%. Detailed demographics of the sample are shown in Table 1.

### 3.2. Measurement

Illegitimate tasks were measured with an 8-item scale developed by Semmer et al., containing 4 items each in two dimensions: unreasonable tasks and unnecessary tasks [20]. The former dimension items start with the introduction, “Do you have work tasks to take care of, which you believe...”, followed by statements such as “…are going too far and should not be expected from you?” The latter dimension items start with the introduction, “Do you have work tasks to take care of, which keep you wondering if...”, followed by statements such as, “…they have to be done at all?” Illegitimate tasks was confirmed as a one-factor construct with unreasonable tasks and unnecessary tasks as two indicators, as such, we similarly regarded illegitimate tasks as a one-factor construct and averaged the scores of all eight items to yield and overall score [24,25,38]. The results of the factor analysis revealed that only one common factor was extracted. The average variance extracted (AVE) equals 0.632 (>0.5), and composite reliability (CR) equals 0.932 (>0.7), indicating a good validity of the scale. The Cronbach’s alpha for the scale was 0.916 in this study.

Job burnout was measured using the MBI-GS scale, an internationally used scale revised by Li et al. based on Chinese organizational contexts [66]. There are five items on the emotional exhaustion dimension, such as “I feel exhausted at the end of the day”; four items on the cynicism dimension, including “I care less and less about whether I am contributing to the work I do”; and six items on the reduced personal accomplishment dimension, such as “I have accomplished a lot of valuable work”. Referring to the practice of related studies, the six items on the reduced personal accomplishment dimension were reverse scored to make the three dimensions consistent in characterizing job burnout. The higher the score is, the higher the degree of job burnout. The results of the factor analysis revealed that three common factors were extracted, corresponding to the three dimensions of job burnout. AVE = 0.636, CR = 0.897, and Cronbach’s alpha = 0.915 for the emotional exhaustion dimension; AVE = 0.638, CR = 0.876, and Cronbach’s alpha = 0.897 for the cynicism dimension; AVE = 0.700, CR = 0.933, and Cronbach’s alpha = 0.920 for the reduced personal accomplishment dimension. These indicators all exceeded the critical values, indicating that the scale has good validity and reliability. A second-order CFA model was constructed for the 15 items of the three dimensions. We used the indicative threshold values for the tests of “close fit” (χ^2^/df ≤ 5; CFI ≥ 0.9; NFI ≥ 0.9; IFI ≥ 0.9; RMSEA ≤ 0.08 suggests an acceptable model–data fit) [67,68]. The indicators obtained from the analysis were χ^2^ = 336.719, df = 87, χ^2^/df = 3.870, RMSEA = 0.079, GFI = 0.910, NFI = 0.934, IFI = 0.950, TLI = 0.940. The results showed an acceptable model fit. Therefore, this study used job burnout as an overall variable for the subsequent statistical analysis and no further subdimensional analysis. The Cronbach’s alpha for the total scale was 0.908 in this study.

Psychological entitlement was measured using the 4-item scale revised by Yam et al., with sample items such as, “I honestly feel I’m just more deserving than others” [69]. The results of the factor analysis revealed that only one common factor was extracted. The AVE = 0.734, and CR = 0.917, indicating a good validity of the scale. The Cronbach’s alpha for the scale was 0.879 in this study.

Collective climate was measured using the 8-item collectivism scale used and validated by Van Hooft et al. [70], with sample items such as, “I make an effort to avoid disagreements with my group members”. The results of the factor analysis revealed that only one common factor was extracted. The AVE = 0.478, and CR = 0.878, indicating an acceptable validity of the scale [71]. The Cronbach’s alpha for the scale was 0.835 in this study. When measuring collective climate, referring to the common practice in the relevant literature, the team members scored the items and then aggregated the individual data to the team level. The results of the aggregation test were as follows: Rwg = 0.93, ICC (1) = 0.34, ICC (2) = 0.72. All values exceeded conventional thresholds (Rwg value exceeded 0.70; ICC (1) value exceeded 0.12; and ICC (2) value exceeded 0.60) [72]). Therefore, the collectivist individual source data can be aggregated to the team level.

In this study, the Likert 5-point scoring method was used for each scale, with illegitimate tasks scored from 1 to 5 in order of frequency from “never” to “always”, job burnout scored from 1 to 5 in order of frequency from “never” to “daily”, and the rest of the scales ranged from “strongly disagree” to “strongly agree”. In addition, we controlled for demographic variables that have been found to be significantly related to job burnout as suggested by Maslach et al. [4], namely gender, age, and education level. Since we conducted a cross-level study, we refer to related studies and include team size and company nature as control variables for the team level [30,72,73]. The possible effects of each control variable were fully considered in this study.

### 3.3. Analytic Methods

Statistical analysis of the data was performed using SPSS 22.0, Mplus 7.4 and HLM 6.08. First, confirmatory factor analysis was performed to examine the discriminant validity of four latent variables; second, descriptive statistics and correlation analysis was conducted on the sample data to understand the distribution of the variables and calculate the Pearson correlation coefficient between variables; and third, we used a cross-level stepwise regression analysis technique to test both the mediating and moderating effects. The “1-1-1” mediation model analysis procedure proposed by Zhang et al. was used to test the mediating effect of psychological entitlement [74]. Regarding the cross-level moderating effect of collective climate, we tested it according to the analysis steps proposed by Aguinis et al. [75]. Furthermore, to test the moderated mediation effect, we estimate two sets of indirect effects at high and low levels of the moderator. Moreover, we constructed 95% CIs for the difference score between these two indirect effects [71].

## 4. Results

### 4.1. Confirmatory Factor Analysis

To examine the discriminant validity of four latent variables, including illegitimate tasks, psychological entitlement, job burnout, and collective climate, several nested structural models were constructed, and confirmatory factor analysis was performed on the sample data. The fit of the nested models is shown in Table 2. The results showed that the 4-factor model had the best fit among the nested models (χ^2^/df = 2.495, RMSEA = 0.057, GFI = 0.942, NFI = 0.947, CFI = 0.968, TLI = 0.960), and each fit indicator met the empirical criteria, so the four main variables in this study can be considered to have good discriminant validity.

### 4.2. Descriptive Statistics and Correlation Analysis

The descriptive statistics and Pearson correlation coefficients are listed in Table 3. Illegitimate tasks were significantly positively correlated with employee psychological entitlement and job burnout (β = 0.303, *p* < 0.01; β = 0.562, *p* < 0.01); employee psychological entitlement was significantly positively correlated with job burnout (β = 0.386, *p* < 0.01); and collective climate was significantly negatively correlated with employee psychological entitlement and job burnout (β = −0.377, *p* < 0.01; β = −0.327, *p* < 0.01). The results preliminarily verified the hypothesis of this study and provided support for subsequent tests.

### 4.3. Hypothesis Testing

#### 4.3.1. Mediating Effects Test

The results are shown in Table 4. First, a null model (Model 1 and Model 4) was established for employee job burnout and psychological entitlement. Second, to distinguish the within-group and between-group variation of illegitimate tasks and psychological entitlement, the two were separately group mean-centered, and the group mean was placed into the intercept term of level 2. As shown in Model 2, the within-group effect (γ_10_ = 0.510, *p* < 0.01) and the between-group effect (γ_01_ = 0.601, *p* < 0.001) of illegitimate tasks on employee job burnout were both positive and significant, assuming that H1 is confirmed. In Model 5, the within-group effect (γ_10_ = 0.324, *p* < 0.001) and the between-group effect (γ_01_ = 0.337, *p* < 0.05) of illegitimate tasks on employees’ psychological entitlement were both positively significant, showing that H2 was confirmed; entering both illegitimate tasks and psychological entitlement into the model, as shown in Model 3, the within-group effect of psychological entitlement on job burnout was significant (γ_20_ = 0.244, *p* < 0.001), while the within-group effect of illegitimate tasks was still significant (γ_10_ = 0.428, *p* < 0.001), but the coefficient was significantly lower than that of Model 2, so it can be considered that psychological entitlement partially mediates the positive effect of illegitimate tasks on employee job burnout.

To test the robustness of the research conclusions, the Monte Carlo method was further used to test the significance of the within-group mediation effect of psychological entitlement. The results found that the indirect effect of psychological entitlement in the relationship between illegitimate tasks and employee job burnout has a 95% confidence interval of [0.044, 0.121], which does not include 0, so it can be considered that the mediating effect of psychological entitlement is significant. In summary, H3 is verified.

#### 4.3.2. The First-Stage Moderated Mediation Effect Test

First, the cross-level moderating effect of a collective climate on the relationship between illegitimate tasks and employee psychological entitlement was tested. As shown in Table 4, in Model 4, ICC (1) = τ_00_/(τ_00_ + σ^2^) = 0.333, indicating that 33.3% of the variance in psychological entitlement can be explained by the between-group variation, necessitating the introduction of variables at the level-2 level. A random intercept fixed-slope model (Model 6) was subsequently constructed and found significant within-group effects of illegitimate tasks on psychological entitlement (γ_10_ = 0.324, *p* < 0.001) and direct cross-level effects of collective climate on psychological entitlement (γ_01_ = −0.379, *p* < 0.01). To further investigate whether the relationship between illegitimate tasks and psychological entitlement has intergroup differences, a random intercept random-slope model (Model 7) was constructed, and it was found that τ_11_ = 0.030 reached a significant level (*p* < 0.01), so the collective climate level-2 variable was further introduced into the equation with the slope as the outcome variable, and a cross-level interaction model (Model 8) was constructed. It was found that the interaction term significantly negatively predicted psychological entitlement (γ_11_ = −0.282, *p* < 0.01), indicating that the collective climate negatively moderates the positive relationship between illegitimate tasks and psychological entitlement. Hypothesis H4 was confirmed.

**Figure 2:**
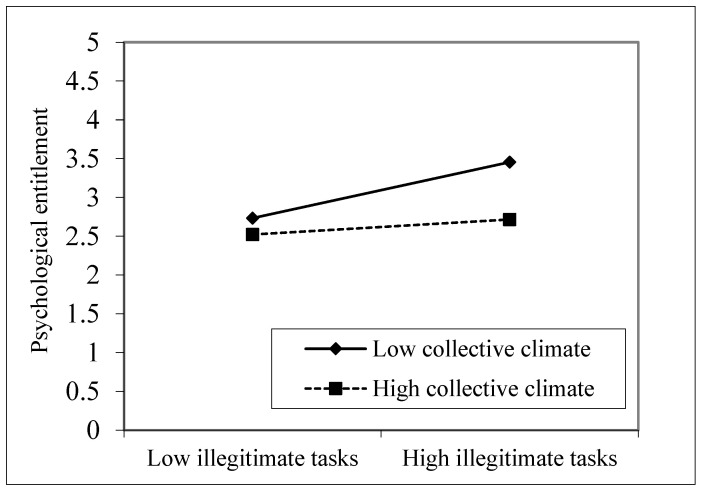
The moderating effect of collective climate on the relationship between illegitimate tasks and psychological entitlement.

To further illustrate the moderating effect, a simple slope analysis was used to draw the moderating effect diagram, as shown in Figure 2. Under a highly collective climate, the regression line of illegitimate tasks on employee psychological entitlement is more gradual, indicating that a collective climate helps to alleviate the stimulating effect of illegitimate tasks on employee psychological entitlement.

The moderating effect of collective climate on the mediating effect of psychological entitlement was tested with the help of Mplus 7.4, and the results are shown in Table 5. In the low collectivism climate, the indirect effect was significant (β = 0.097, *p* < 0.001, CI = [0.043, 0.151]), while in the high collectivism climate, the indirect effect was still significant but obviously weakened (β = 0.052, *p* < 0.01, CI = [0.013, 0.091]). Additionally, the difference between the two was significant (β = −0.045, *p* < 0.05, CI = [−0.085, −0.004]). To test the robustness of the results, the Monte Carlo method was used to verify the indirect effect difference between high and low collective climates. With the help of R software, 20,000 repeated samplings were set up, and the results revealed that the 95% confidence interval for the indirect effect difference was [−0.236, −0.017]. Thus, it can be concluded that collective climate negatively moderates the mediating role of psychological entitlement in the relationship between illegitimate tasks and job burnout. H5 was verified.

## 5. Discussion

The continuous increase in work pressure has become a common pain point for working people. In 2019, the National Health Commission of the People’s Republic of China issued the “Healthy China Action Plan (2019–2030)”, emphasizing mental health, occupational health, and other issues and encouraging various organizations to adopt comprehensive measures to reduce or eliminate work stress. Since then, it has become a topic of people’s livelihoods that has been widely considered by many in all walks of life in China. Scholars have conducted extensive discussions on tracing the source of work stress and clarifying its impact mechanism. In this context, illegitimate tasks have attracted increasing attention. Relevant studies have pointed out that illegitimate tasks have adverse effects on employees’ emotions, cognition, motivation, work attitudes, work behaviors, physical and mental health, and work–family relationships [24,34]. This study provides insight into the impact of illegitimate tasks on job burnout, enriches the research on the negative workplace effect of illegitimate tasks, and for the first time verifies the findings of Semmer et al. [24] and Munir et al. [26] in a Chinese cultural context, confirming that job burnout is one of the negative outcomes of illegitimate tasks and contributing to the identification of the antecedents of job burnout.

Previous studies on illegitimate tasks and employee burnout have been limited to demonstrating a correlation between the two [21,24] or have focused on the mediating role of offensive feeling, organizational justice, anger and role conflict [23,26]. Few studies have gone beyond the perspective of emotional role paths or cognitive role paths to focus on the possible role of psychological entitlement, which is a psychological characteristic widely present in the workplace. Faced with illegitimate tasks, the positive self-concept of employees is undeservedly harmed, and the lack of reciprocal rewards for their efforts will stimulate employees to develop psychological entitlement [76]. Employees with high psychological entitlement will develop unrealistic expectations and self-service attribution bias, resulting in increased demands for psychological resources, leading to job burnout [77]. Based on these inferences, this study verifies the positive relationship between illegitimate tasks and employee psychological entitlement, which provides a new perspective on the role of illegitimate tasks in triggering negative workplace effects. Moreover, it helps to better explain the causes of burnout while enriching the research related to psychological entitlement.

Collective culture emphasizes the identity of the individual in the collective and the resulting obligations and responsibilities, advocating the spirit of self-sacrifice that can be made for the benefit of the organization [58]. Culture lays the foundation of individual values through socialization processes, shapes individual thinking patterns, and plays an important role in individual information processing; that is, culture influences the formation process of individual concepts, perceptions, and judgments by affecting internal mental activities and ultimately has an impact on individual behavior [32]. Collective culture specifically manifests itself in organizations and work teams as a collective climate. Similar to the finding of Li and Xu regarding the moderating effect of collective climate [58], this study found that employees’ negative coping responses to illegitimate tasks had different outcomes during ongoing cognitive processing due to the influence of collective climate. A high collective climate that advocates selfless contributions to the organization helps ameliorate employees’ perception of injustice formed by illegitimate tasks, thereby alleviating their psychological entitlement, and the effect of illegitimate tasks on employee burnout is further attenuated as a result.

### 5.1. Theoretical Implications

First, this study focuses on the problem of job burnout that restricts organizational development and affects employees’ occupational health, verifies the stimulating effect of illegitimate tasks on employee job burnout, improves the research on the negative workplace effects of illegitimate tasks, and provides a new way of thinking about the issue of employee job burnout that has received much attention in recent years. Second, this study reveals the intrinsic mechanism of job burnout induced by illegitimate tasks. Combining justice theory and JD-R theory, this study verifies the mediating role of employee psychological entitlement in the process of illegitimate tasks acting on employee burnout, thereby enriching the study of employee psychological entitlement from the perspective of JD-R theory. Third, this study builds a cross-level moderated mediation theoretical model and examines the moderating effect of a collective climate. While rooted in specific cultural contexts and promoting the development of localized research, these findings help reveal the boundary conditions of the influence process of illegitimate tasks on employees and improve the study of the influential mechanism of illegitimate tasks.

### 5.2. Practical Implications

This study focuses on the new workplace stressor of illegitimate tasks, explores the specific mechanisms of their effects on employee job burnout and proposes the following management insights:

(1) In management practice, organizations and managers should face the negative impact of illegitimate tasks on the development of employees and organizations. On the one hand, organizations should focus on guiding managers to prevent them from issuing illegitimate tasks. Managers’ awareness of illegitimate tasks can be enhanced through special training to promote the simultaneous improvement of their management skills and professional ethics. Managers should use their power carefully when assigning tasks and directing work, clarify the role expectations of their subordinates, and stop intentionally or unintentionally bringing illegitimate tasks to their employees.
(2) Managers should pay careful attention to the psychological state of employees. When employees are found to have excessive levels of psychological entitlement, managers should actively reflect on whether they have been assigned illegitimate tasks that are causing them to be mismatched with their jobs and resulting in the nonoptimal allocation and utilization of resources. Correct the mistakes, if any, and keep the good record if none has been committed. At the same time, organizations should channel the negative emotions of employees with such problems through seminars and psychological counseling, guide them to evaluate things objectively and make up for the psychological resources to prevent an increase in employee job burnout.
(3) Organizations should pay attention to the positive effects that a collective climate may have on a work team. When recruiting employees, priority should be given to hiring employees with higher collectivist tendencies. In addition, managers should focus on building a collective culture. Through creating exemplary figures, theme education, and other cultural activities to promote and advance collectivism, coupled with a corresponding incentive system, managers can help promote a collective climate among the work team members, thus alleviating employees’ negative perceptions and judgments about their jobs due to excessive focus on personal gains and losses and manifesting job burnout.

### 5.3. Limitations and Future Studies

Due to the constraints of time and resources, this study has some limitations that need to be improved upon in a follow-up study:

(1) The effect of illegitimate tasks on employee psychological entitlement and job burnout has a time-lag effect. This study uses cross-sectional data, which makes it difficult to fully clarify the rule of the effect. Future research can consider deepening the research design, collecting data at a longitudinal multitime point, and increasing the sample data sources through mutual evaluations of managers and employees to reveal the relevant influence mechanism more scientifically and accurately.
(2) For the measurement of the study variables, this study used well-established measurement scales based on Western cultural contexts. Although the scales are widely used and proven to have good reliability, their full applicability to the Chinese organizational culture remains to be explored, especially for the illegitimate task measurement scale. The differences between Chinese and Western cultures and work philosophies may bring about different interpretations and orientations of illegitimate tasks among employees. Future studies may consider revising or redeveloping the illegitimate task measurement scale based on the Chinese cultural context. Another limitation of the study is failing to control for employees who were suffering from burnout, and testing other factors of burnout and entitlement. In our future studies, these phenomena will be considered.
(3) Based on justice theory and the JD-R model, this study introduced psychological entitlement as a mediating variable and found that psychological entitlement only played a partial mediating role. Future research can consider a more in-depth exploration of other mediating variables; for example, it can systematically construct a dual emotion-cognition channel model based on the cognitive-affective system theory of personality to compare the cognitive and emotional paths of the effect of illegitimate tasks. In addition, considering the complexity of individual responses to illegitimate tasks, boundary conditions such as individual traits, in addition to collective climate, remain to be explored.

## 6. Conclusions

Identifying work stressors and clarifying the negative effects caused by them to alleviate work stress and promote employees’ occupational health is one of the hot topics in the field of organizational behavior and human resource management in the post-pandemic era. Through theoretical derivation and empirical analysis, this study explores the mediating paths and boundary conditions of illegitimate tasks to employee job burnout. The study reveals the antecedents of burnout from the perspective of job tasks and psychological entitlement, offering practical insight into the mechanism of illegitimate tasks on employee job burnout. As investigated in the current research, illegitimate tasks stimulate the formation of psychological entitlement and lead to employee burnout. Psychological entitlement partially mediates the positive effect of illegitimate tasks on employee burnout. A collective climate can buffer the stimulating effect of illegitimate tasks on employees’ psychological entitlement and weaken the mediating effect of psychological entitlement between illegitimate tasks and employee job burnout. This study provides followers with the cues regarding negative impact of illegitimate tasks, advocates followers to explore the effects of illegitimate tasks on employees from both emotional and cognitive paths, and recommends that organizations develop a collective climate to reduce employees’ psychological entitlement and job burnout for the steady development of the enterprise and employees’ occupational health.

## Figures and Tables

**Table 1:** The demographics of the sample (*N* = 459).

Demographic Variables	Categories	Number of Participants	Percentage (%)
Gender	Male	205	44.66
	Female	254	55.34
Age	Under 20 years old	1	0.22
	21–25 years old	87	18.95
	26–30 years old	110	23.97
	31–35 years old	99	21.57
	36–40 years old	61	13.29
	Over 40 years old	101	22.00
Educational level	Senior high school (technical secondary school) and below	56	12.20
	Junior college	89	19.39
	Undergraduate College	264	57.52
	Postgraduate	50	10.89
Working years	Under 1 year	64	13.94
	1–2 years	72	15.69
	2–3 years	35	7.62
	3–5 years	86	18.74
	More than 5 years	202	44.01

**Table 2:** Results of the confirmatory factor analysis.

Model	χ^2^	df	χ^2^/df	RMSEA	GFI	NFI	CFI	TLI
1-Factor: IT + PE + JB + CC	1895.480	90	21.061	0.209	0.550	0.525	0.535	0.458
2-Factor: IT + CC, PE + JB	1301.753	89	14.626	0.172	0.685	0.674	0.688	0.632
3-Factor: IT + PE, JB, CC	1093.732	87	12.572	0.159	0.697	0.726	0.741	0.687
3-Factor: IT, PE, JB + CC	640.944	87	7.367	0.118	0.833	0.839	0.857	0.828
3-Factor: IT, PE + JB, CC	598.860	87	6.883	0.113	0.847	0.850	0.868	0.841
4-Factor: IT, PE, JB, CC	209.551	84	2.495	0.057	0.942	0.947	0.968	0.960

Note: IT = illegitimate tasks; PE = psychological entitlement; JB = job burnout; CC = collective climate.

**Table 3:** Descriptive statistics and correlation coefficient matrix of variables.

Variable	Mean	SD	1	2	3	4	5	6	7	8	9
1. Gender	1.553	0.498	-								
2. Age	3.948	1.426	0.016	-							
3. Educational level	2.671	0.827	−0.093 *	−0.233 **	-						
4. Company nature	2.237	1.559	0.193 **	0.150 **	0.150 **	-					
5. Working years	3.632	1.506	0.048	0.593 **	−0.096 *	0.228 **	-				
6. IT	2.376	0.741	−0.067	0.001	0.198 **	0.077	0.161 **	-			
7. PE	2.820	0.849	−0.101 *	−0.045	0.098 *	−0.140 **	0.098 *	0.303 **	-		
8. JB	2.231	0.718	0.034	−0.125 **	0.219 **	0.110 *	0.051	0.562 **	0.386 **	-	
9. CC	3.669	0.632	0.141 **	0.009	−0.025	0.106 *	−0.085	−0.198 **	−0.377 ^**^	−0.327 **	-

Note: *N* (employees) = 459; ** *p* < 0.01, * *p* < 0.05; IT = illegitimate tasks; PE = psychological entitlement; JB = job burnout; CC = collective climate.

**Table 4:** The results of cross-level regression analysis for mediating effect.

Variable		JB			PE				
		Model 1	Model 2	Model 3	Model 4	Model 5	Model 6	Model 7	Model 8
Intercept		2.278 ***	0.731 *	0.287	3.013 ***	2.144 ***	2.938 ***	2.881 ***	2.856 ***
Level-1 variable	Gender	0.037	0.102	0.106 *	−0.063	−0.013	−0.004	0.024	0.035
	Age	−0.111 ***	−0.076 **	−0.072 **	−0.030	−0.009	−0.017	0.008	0.009
	Educational level	0.092 ***	0.027	0.007	0.116 ***	0.076 **	0.087 **	0.064 *	0.065 *
	IT		0.510 **	0.428 ***		0.324 ***	0.324 ***	0.317 ***	0.309 ***
	PE			0.244***					
Level-2 variable	Company nature	0.049	0.030	0.058 *	−0.105 **	−0.121 **	−0.088 **	−0.087 **	−0.092 **
	Team size	−0.020	0.010	0.014	−0.030	−0.014	−0.030	−0.029	−0.027
	Group mean of IT		0.601 ***	0.525 ***		0.337 *			
	Group mean of PE			0.207 **					
	CC						−0.379 **	−0.436 ***	−0.377 **
	IT * CC								−0.282 **
σ^2^		0.405	0.292	0.257	0.465	0.413	0.418	0.382	0.378
τ_00_		0.091 ***	0.047 **	0.041 ***	0.232 ***	0.227 ***	0.221 ***	0.604 **	0.663 **
τ_11_								0.030 **	0.026 *

Note: *N* (employees) = 459, *N* (teams) = 89; All coefficients are estimates of the fixed effect under robust standard error (γ). σ^2^ is the residual of level-1, τ_00_ is the intercept residual of level-2, τ_11_ is the slope residual of level-2; *** *p* < 0.001, ** *p* < 0.01, * *p* < 0.05; IT = illegitimate tasks; PE = psychological entitlement; JB = job burnout; CC = collective climate.

**Table 5:** The testing results of the moderated mediation effect.

Moderating Variable	IT (X)→PE (M)→JB (Y)			
	Indirect Effect Estimate	*p*	LLCI	ULCI
Low CC (−1SD)	0.097	0.000	0.043	0.151
High CC (+1SD)	0.052	0.009	0.013	0.091
High-Low CC difference	−0.045	0.031	−0.085	−0.004

Note: IT = illegitimate tasks; PE = psychological entitlement; JB = job burnout; CC = collective climate.

## Data Availability

The data will be made available on request from the corresponding author.
